# Extended spectrum and metalo beta-lactamase producing airborne *Pseudomonas aeruginosa* and *Acinetobacter baumanii* in restricted settings of a referral hospital: a neglected condition

**DOI:** 10.1186/s13756-017-0266-0

**Published:** 2017-10-23

**Authors:** Fithamlak Bisetegen Solomon, Fiseha Wadilo, Efrata Girma Tufa, Meseret Mitiku

**Affiliations:** 1 School of Medicine, Wolaita Sodo University, PO box 138, Sodo, Ethiopia; 2 Wolaita Sodo University, School of Medicine, Sodo, Ethiopia; 3 Department of medical laboratory, Wolaita Sodo University, Sodo, Ethiopia; 4 College of health science and medicine, school of medicine, MaddaWalabu University, Bale Goba, Ethiopia

**Keywords:** Antibiotic resistance, ESBL, MBL, *P. aeruginosa*, *A. Baumannii*, MDR, Airborne

## Abstract

**Background:**

Frequently encountered multidrug-resistant bacterial isolates of *P. aeruginosa* and *A. baumannii* are common and prevalent in a hospital environment. The aim of this study was to determine the prevalence and pattern of antibiotic resistance, extended spectrum and metallo beta-lactamase producing *P. aeruginosa* and *A. baumannii* isolates from restricted settings of indoor air hospital environment.

**Methods:**

A hospital-based cross-sectional study was conducted in Wolaita Sodo University Teaching and referral Hospital, Ethiopia from December 1/2015 to April 30/2015. The Air samples were collected from delivery room, intensive care unit and operation theatre of the hospital by active, Anderson six slate sampler technique during the first week of the months, twice a week during Monday’s and Friday’s. Standard microbiological procedures were followed to isolate *P. aeruginosa* and *A. baumannii.* Susceptibility testing was performed on isolates using the Kirby-Bauer disk diffusion technique. Extended spectrum beta lactamase production was detected by double disc synergy test and Imipenem-resistant isolates were screened for producing Metallo-beta lactamase.

**Results:**

A total number of 216 indoor air samples were collected from the delivery room, intensive care unit, and operation room. Correspondingly, 43 *A. baumannii* isolates were identified (13 from delivery room, 21 from intensive care unit and 9 from operation room). Likewise 24 *P. aeruginosa* isolates were obtained (4 from delivery room, 13 from intensive care unit and 7 from operation room). Extended spectrum beta lactamase and metalo-beta lactamase production were observed in 24 (55.8%) and 13 (30.2%) isolates of *A. baumannii* respectively, whereas *P. aeruginosa* showed 15 (62.5%) extended spectrum beta lactamase and 9 (37.5%) metallo-beta lactamase production.

**Conclusions:**

Extended spectrum beta lactamase and metallo-beta lactamase producing bacteria in hospital air is a new dimension for specific setting of the study area where antimicrobial resistance is increasing and surgical site infection is prevalent. So, identification of these microorganisms has a great role in reducing the burden of antibiotic resistance and could also provide a significant input for framing hospital infection control policies.

## Background

Airborne microorganisms could cause respiratory disorders, severe infections, hypersensitivity pneumonitis and toxic reactions [1]. Frequently encountered multidrug-resistant (MDR) bacterial isolates like Ceftazidime-resistant *Pseudomonas aeruginosa* and Imipenem*-*resistant *Acinetobacter baumannii* are common and prevalent in a hospital environment [2–5].

Multidrug-resistant *P. aeruginosa* is inherently resistant to many drug classes and is able to acquire resistance to all effective antimicrobial drugs [6]. MDR *P. aeruginosa* elaborates inactivating enzymes that make beta-lactams and carbapenems ineffective, such as extended spectrum beta lactamases (ESBLs) and metallo-β-lactamases (MBLs) [7].

*A. baumannii* also remain problematic because of its high intrinsic resistance to a wide variety of antimicrobial agents. Moreover, the ability of resistant strains of *A. baumannii* to survive for prolonged periods in the hospital environment contributes significantly to antimicrobial resistance, thereby posing a difficult challenge for infection control [8, 9]. Carbapenems used to be the drugs of choice for treating burn infections caused by *A. baumannii* strains. Consequently, due to selective pressure on carbapenems and the increased use of this antibiotic, carbapenem-resistant *A. baumannii* has emerged. This problem worsens in cases of MBL production when the drug of last choice, carbapenems, is inactive [10].

The uncontrolled movement of air in and out of the hospital environment makes the bacterial persistence worse since these infectious microorganisms may spread easily into the environment through sneezing, coughing, talking and contact with hospital materials. It can affect patients admitted to rooms in which the prior occupants tested positive for a pathogen and also other patients in the facility [11, 12].

Therefore, the main objective of this study was to determine the prevalence and pattern of ESBL and MBL producing *P. aeruginosa* and *A. baumannii* from hospital indoor air of Wolaita Sodo University Teaching and Referral Hospital (WSUTRH).

## Methods

### Study area

The study was conducted at Wolaita Sodo University Teaching and Referral Hospital (WSUTRH), Sodo, located South Central Ethiopia. It is serving people in catchment’s area of 2 million people. The hospital has 320 beds for inpatient service which are on medical, pediatrics, surgical, intensive care unit, gynecology and obstetrics wards.

### Study design and period

A hospital based cross sectional study was conducted to determine the prevalence and pattern of antibiotic resistance, extended spectrum and metallo beta-lactamase producing *P. aeruginosa* and *A. baumannii* isolates from restricted settings of indoor air hospital environment. The study was undertaken from December 1, 2015 to April 30, 2016 in WSUTRH.

### Sampling techniques

The Air samples were collected during the first week of the months, twice a week during Monday’s and Friday’s. All microbiological procedures were conducted in Wolaita Sodo University microbiology laboratory which is an accredited laboratory with bio-safety cabinet two and vitek 2 microbiology apparatus. The laboratory built independently 5 km far from the clinical departments where air samples were conducted.

### Active air sampling

Active air sampler, Anderson six state cascade impactor, which sucks 28.3 l of air per minute, was used and the Petridish was placed in the impactor for 5 minutes [13]. After that the Petridish was shipped to Wolaita Sodo university microbiology laboratory. Petri dishes were labeled with sample number, hospital ward, date and time (hour, minute and second) of sample collection.

Three agar plates were placed at various distances in each of the selected wards with five meter apart. Self-contamination was prevented by wearing sterile surgical gloves, mouth masks, and protective gown.

### Processing of specimens and preliminary identification

Following collection, colonies on tryptic soya agar were inoculated into MacConkey agar, and blood agar plates. The inoculated plates were incubated at 35 °C for 24–48 h. Then the growth was inspected to identify the bacteria.

*P. aeruginosa* isolates were presumptively identified by gram staining, colony morphology, pigment formation, mucoid, haemolysis on blood agar, positive oxidase test, grape-like odour, growth at 42 °C on nutrient agar, and positive motility [14].

Genus *Acinetobacter* was identified by Gram staining, cell and colony morphology, positive catalase test, negative oxidase test and absence of motility. Suspected *A. baumanii* isolates were confirmed by API-20 NE kit (biomerieux, France) system.

### Antibiotic susceptibility testing

The drug susceptibility testing of the isolates was done by Kirby-Bauer disc diffusion method [15] following Clinical Laboratory Standards Institute (CLSI) guide lines. The grades of susceptibility pattern were recognized as sensitive, intermediate and resistant by comparison of the zone of inhibition as indicated by CLSI, 2014 [16]. Intermediate isolates were taken as sensitive for the purpose of this study. The antibiotic discs were obtained from Oxoid, England, with the following concentrations: amikacin (30 μg), cefotaxime (30 μg), cefepime (30 μg), azetronam (30 μg) amoxicillin-clavulanic acid (30 μg), ceftazidime (30 μg), ceftriaxone (30 μg), ciprofloxacin (10 μg), meropenem (10 μg), gentamicin (10 μg), imipenem (10 μg), trimethoprim-sulphamethoxazole (25/1.25 μg). Antibiotics were selected based on local availability, their effectiveness, guideline provided by CLSI and from literatures.

### Phenotypic detection of extended spectrum beta-lactamase producing bacteria

Extended spectrum beta-lactamase (ESBL) production was detected by double disc synergy test (DDST) [17]. Accordingly, 3–5 selected colonies were taken from a pure culture and transferred to a tube containing 5 ml sterile nutrient broth and mixed gently until a homogenous suspension was formed. The suspension was incubated for 4–6 h at 37 °C until the turbidity was matched with the 0.5 McFarland standards. A sterile cotton swab was then used to distribute the bacteria evenly over the entire surface of Mueller Hinton agar (Oxoid, England).

Amoxicillin-clavulanic acid disc was placed in the center of the plate whereas ceftriaxone, ceftazidime and cefotaxime (30 μg each) discs were placed at a distance of 20 mm (center to center) from the amoxicillin-clavulanic acid disk. The plates were then incubated at 37 °C for 24 h and results were read. Enhancement of zone of inhibition of the cephalosporin disc towards clavulanic acid containing disc was inferred as synergy and the strain considered as ESBL producer.

### Phenotypic detection of metalo-beta lactamase producing bacteria

Imipenem-resistant isolates were screened for producing MBL. The double disk method was used to detect this enzyme. Colonies from overnight cultures on blood agar plates were suspended in Mueller-Hinton broth and the turbidity standardized to equal that of a bacterial concentration of 1:100 suspensions of the 0.5 McFarland standards. Then the suspension was streaked onto Mueller-Hinton agar plates (Hi Media, Mumbai, India). A disc of Imipenem alone (10 μg) and Imipenem (10 μg) in combination with EDTA (750 μg/disc) was placed at the distance of 20 mm (centre to centre). After overnight incubation at 35 °C, a ≥ 7 mm increase in the inhibition zone of diameter around Imipenem-EDTA discs, as compared to imipenem discs alone, interpreted as indicative of MBL production [18].

### Operational definitions

**MDR** was defined as acquired non-susceptibility to at least one agent in three or more antimicrobial categories.

**Pan resistance**-resistance for all antibiotics tested.

**High MDR**: resistance rate of the isolates for more than 60% of the antibiotics.

### Quality controls

Standard operating procedures were prepared and followed from sample collection to reporting. Culture medias were prepared based on the manufacturers’ instruction then the sterility was checked by incubating 5% of the batch at 35-37 °C for overnight and observing bacterial growth. Those Media which showed growth were discarded. Anderson air sampler was handled by environmental microbiologist and as per the manufacturer’s instruction.

*Escherichia coli* ATCC 25922 and *Pseudomonas aeruginosa* ATCC 27853 were used as control strains.

### Data analysis

Statistical analysis was performed by using SPSS version 20 software program and descriptive statistics were used.

## Results

### Microbial load of hospital wards

A total of 216 indoor samples were collected from intensive care unit (ICU), delivery room (DR) and operation room (OR). Correspondingly, 67 isolates (43 *A. baumannii* and 24 *P. aeruginosa*) were obtained with an overall isolation rate of 31% (67/216). Of those isolates, the highest rate (50.7%) was identified from ICU, whereas the lowest rate (23.9%) was from OR (Table 1).

**Table 1:** Distribution of airway *A. baumannii* and *P. aeruginosa* isolates in wards of WSUTRH

Wards	*A. baumannii n* = 43	*P. aeruginosa n* = 24	Total isolates *n* = 67
Delivery room	13	4	17
Intensive care unit	21	13	34
Operation room	9	7	16

### Antibiotic resistance profile of air-borne bacterial pathogens

*A. baumannii* showed a high level of resistance, i.e. >80%, for each of trimethoprim-sulfamethoxazole, cefepime, ciprofloxacin and ceftriaxone antibiotics whereas *P.aeruginosa* showed a high resistance percentage for trimethoprim-sulfamethoxazole, ciprofloxacin and ceftriaxone antibiotics with the rate of 88.2%, 83.3, and 79.1% respectively (Table 2).

**Table 2:** Antibiotic resistance profile of air-borne *A. baumannii* and *P. aeruginosa*

Antibiotics	*A. baumannii* (*n* = 43) No (%)	*P.aeruginosa* (*n* = 24) No (%)
Amikacin	30 (69.8)	6 (25)
Cefotaxime	32 (74.4%)	17 (70.8)
Cefepime	38 (88.4%)	14 (58.3)
Ceftazidime	28 (65.1)	7 (29.1)
Ciprofloxacin	38 (88.4%)	20 (83.3)
Gentamicine	33 (76.7)	19 (79.1)
Ceftriaxone	36 (83.7)	19 (79.1)
Aztreonam	19 (44.2)	14 (58.3)
Meropenem	13 (30.2)	10 (41.7)
Imipenem	16 (37.2)	10 (41.7)
Trimethoprim-Sulfamethoxazole	40 (93.0)	21(87.5)

### ESBL and MBL production by *A. baumannii*

From the total isolates of *A. baumannii*, 38 (88.4%) of them showed resistance to at least one of the third generation cephalosporins (3GC). ESBL and MBL production were observed in 24(55.8%) and 13 (30.2%) of the isolates respectively. Coexistence of both ESBL and MBL producers was seen in 5(11.6%) isolates of *A. baumannii* (Table 3)*.*

**Table 3:** ESBL and MBL producing airway *A.baumannii* isolates in restricted settings of the Hospital

Number of resistance isolates (%)			
Antibiotics	Total isolates *n* = 43	ESBL producer *n* = 24	MBL producer *n* = 13
Ceftazidime	28 (65.1)	19 (79.2)	8 (61.5)
Ceftriaxone	36 (83.7)	22 (91.7)	10 (76.9)
Cefepime	38 (88.4)	23 (95.8)	11 (84.6)
Cefotaxime	32 (74.4)	20 (83.3)	9 (69.2)
Aztreonam	19 (44.2)	21 (87.5)	12 (92.3)
Impeniem	16 (37.2)	5 (20.8)	13 (100)
Meropenem	13 (30.2)	3 (12.5)	13 (100)

### ESBL and MBL production by *P. aeruginosa*

Out of 24 isolates of *P. aeruginosa,* 15 (62.5%) were found to become ESBL producers. Metalo-beta-lactamase production was observed in 9 (37.5%) of *P. aeruginosa* isolates. Co-occurrence of both ESBL and MBL producers were seen in 5 (20.8%) isolates (Table 4).

**Table 4:** ESBL and MBL producing airway *P. aeruginosa* isolates in intensive care unit of the hospital

Number of resistance isolates (%)			
Antibiotics	Total isolates *n* = 24	ESBL producer *n* = 15	MBL producer *n* = 9
Ceftazidime	7 (29.1)	11 (73.3)	7 (77.8)
Ceftriaxone	19 (79.1)	13 (86.7)	8 (88.9)
Cefipime	14 (58.3)	10 (66.7)	5 (55.6)
Cefotaxime	17 (70.8)	11 (73.3)	8 (77.8)
Aztreonam	14 (58.3)	10 (66.7)	9 (100)
Impenem	10 (41.7)	5 (50.0)	9 (100)
Meropenem	10 (41.7)	3 (20.0)	9 (100)

### MDR patterns of aerosol *A. baumannii* and *P.aeruginosa*

A total of 35 (81.4%) *A. baumannii* isolates were found out to be multi-drug resistant. Moreover, 7 (16.3%) of the isolates were pan-drug resistant. Likewise about 20 (83.3%) *P. aeruginosa* isolates were multi-drug resistant with 5 (20.8%) of them pan-drug resistant isolates (Table 5).

**Table 5:** Antibiogram of air-borne *A. baumannii* and *P. aeruginosa* isolates

Bacteria	Quantity	Resistance pattern	Frequency	Class
*P.aeruginosa* *n* = 24	Max	TMP-SXT, CIP, GEN, CRO, CTX, ATM, FEP, IMP, MEM, CAZ, AMK	5	6
		TMP-SXT, CIP, GEN, CRO, CTX, ATM, FEP, IMP, MEM, CAZ	2	6
		TMP-SXT, CIP, GEN, CRO, CTX, ATM, FEP, IMP, MEM	2	6
		TMP-SXT,CIP, GEN, CRO, CTX, FEP, IMP, MEM,AMK	1	5
		TMP-SXT, CIP, GEN, CRO, CTX, ATM, FEP	4	5
		TMP-SXT, CIP, GEN, CRO, CTX	3	4
		TMP-SXT, CIP, GEN, CRO, ATM	1	4
		TMP-SXT, CIP, GEN	1	3
	Min	TMP-SXT, CIP, CRO	1	3
*A. baumannii* *n* = 43	Max	TMP-SXT, CIP, FEP, CRO, GEN, CTX, AMK, CAZ, ATM, IMP, MEM	7	6
		TMP-SXT, CIP, FEP, CRO, GEN, CTX, AMK, CAZ, ATM, IMP	3	6
		TMP-SXT, CIP, FEP, CRO, GEN, CTX, AMK, CAZ, IMP, MEM	3	5
		TMP-SXT, CIP, FEP, CRO, GEN, CTX, AMK, CAZ, ATM	6	5
		TMP-SXT, CIP, FEP, CRO, GEN, CTX, AMK, CAZ	6	4
		TMP-SXT, CIP, FEP, CRO, GEN, CTX, AMK	2	4
		TMP-SXT, CIP, FEP, CRO, GEN, CTX	2	4
		TMP-SXT, CIP, FEP, CRO	4	3
	Min	TMP-SXT, CIP, FEP	2	3

Key: AMK-Amikacin, CTX-Cefotaxime, FEP-cefepime CAZ-Ceftazidime, CIP-Ciprofloxacin GEN-Gentamicine CRO-Ceftriaxone, ATM-Aztreonam, MEM-Meropenem, IMP-Imipenem, TMP-SXT-Trimethoprime-Sulphamethoxazole

## Discussion

Several studies have documented extensive contamination by *Acinetobacter spp*. of the environment, including respirators and air samples, in the vicinity of infected or colonized patients [19]. In an outbreak of infection with Multi-resistant *Acinetobacter spp*. extensive contamination of the environment, including air was found [19].

The presence of *A.baumannii* as bioaerosols in this study could be supported by its higher survival ability (3 days to 11 months) in the environment and its disinfectant resistance. As the best of the investigators knowledge, this is the first finding of *A.baumannii* in Hospital air in Ethiopian setup. But our finding was corroborated with previous reports in Taiwan [20], Iran [21] and Nepal [22].

High percentage of antibiotic resistance, more than 80%, *A. baumannii* isolates were detected for trimethoprim-sulfamethoxazole, ciprofloxacin, cefepime and ceftriaxone in this study which is corroborated with findings of previous reports in Iran [23, 24], Turkey [25] and Italy hospital intensive care units [26]. A study in Romania reported highly resistant *A. baumannii* isolates with 75% resistance for ceftriaxone, ceftazidime, gentamicin and kanamycin antibiotics each [27] and a study conducted in Ethiopia also revealed 100% and 88% resistant Ciprofloxacin and Gentamicin *A.baumannii* from environmental isolates respectively [28]. Ciprofloxacin resistant, 86.5% *A.baumannii* isolates were also detected in clinical and environmental isolates in Brazil [29] and 92.2% TMP-SXT resistant isolates were also identified in hospital waste effluent in Denmark [30]. Similarly, high antibiotic resistance percentage were also found in Bangladesh from isolates collected from endotracheal tube with 100% resistance for ceftriaxone and gentamicin, and 66.7% for amikacin and impenem 66.7% [31].

Meropenem and imipenem depicted 30.2% and 37.2% resistance *A. baumannii* in the current study which is in harmony with previous findings of 30.2% Meropenem resistance in India [32], 33.3% and 28.1% imipenem resistance in Egypt [33] and Brazil [29] respectively but much lower than 87.7% and 95% resistance reported for both antibiotics in Turkey [34] respectively which could be due to difference in availability and prescribing pattern of antibiotics where these antibiotics were introduced in our country recently.

ESBLs were reported in the species belonging to the genera of *Enterobacter* and *Klebsiella* isolated from the air of hospital associated environment. 55.8% of *A. baumannii* isolates were ESBL producing. This finding is higher than 21% ESBL production rate reported in Tehran [35] and 28% in India [36].

MBL producer *A. baumannii* rate identified in this study (30.2%) was lower than 48% reported in India [37] and 81.48% reported in environmental isolates in Egypt [38], which could be explained by difference in samples, and reduced selective pressure of *Acinetobacter* for imipenem and meropenem antibiotics in our country setups.

Generally *A.baumannii* showed the highest percentage of resistance for most antibiotics tested, this could possibly be due to the bacterial ability to resist many antibiotics and disinfectant or could possibly be due to selective pressure or abusing of the drugs in the hospital.

*P. aeruginosa* associated infection is a recognized public health threat often acquired from the hospital environment. It is not only an important cause of morbidity but also increases the stay of the patient in the hospital and increases the cost of treatment [39]. The isolation of epidemic *P. aeruginosa* from room air in the presence of patients increases the possibility that there may be airborne spread of epidemic *P. aeruginosa* strains between patients [40].

The antibiotic susceptibility pattern of environmental isolates of *P. aeruginosa* is mostly overlooked and rarely reported. A few reports available on susceptibility pattern of *P. aeruginosa* suggest significant resistance to a variety of antibacterial agents. In this study, high rate (> 60%) of antibiotic resistant *P.aeruginosa* isolates were observed for amikacin, cefotaxime, cefepime, ceftazidime, ciprofloxacin, gentamicin and trimethoprim-sulphamethoxazole. This finding is corroborated with previous study from environmental isolates in Egypt where isolates from the hospital environment have showed more antibiotic resistance than the clinical isolates with rate of resistance 100% for cefotaxime, 92% for ceftriaxone, 85% for gentamicin, 85%, and 62% for ciprofloxacin [41].

A previous study conducted in Ethiopia revealed high antibiotic resistant *P.aeruginosa* isolates in hospital environment. Indoor air pseudomonas species were also showed significant percentage of resistance for Gentamicin (73.7%) and Ciprofloxacin (78.9%) [42]. Higher levels of *P.aeruginosa* resistance to trimethoprim-sulfamethoxazole, gentamicin and ceftriaxone in the present study is comparable with the study conducted in Ethiopia where 95.1% to trimethoprim-sulphametoxazole, 62% to gentamicin, and 58% to ceftriaxone resistance revealed [43].

The rate (41.7%) of resistance of the *P.aeruginosa* isolates to imipenem seen in this study is higher than 18and 18.9% reported in India [44, 45]. Ceftazidime resistance (29.1%), *P.aeruginosa* isolates in this study is different from the previous reported findings in Nigeria 34.6% [46] and India 36% [44]. Variation of resistance across different studies could be due to availability of antibiotics at the hospital as well as community level, type of patients, number of samples, and genotypic resistance mechanisms.

ESBL producing *P.aeruginosa* isolates were mostly detected in clinical isolates in hospital setup according to the university hospital microbiology report. ESBL producing *P.aeruginosa* isolates detected in this study corroborated with a study in Egypt where 95% of *P.aeruginosa* isolates were beta-lactamase producers [6] and all isolates from surface water were ESBL producer in study conducted by Nasereen et al. [47].

In our finding, 37.5% MBL production by *P. aeruginosa* was observed. A study conducted in India, Brazil and Iran revealed 32.9%, 30%, 48.3% MBL production respectively [48–50]; however those studies used clinical specimen. These *P. aeruginosa* isolates could be causes for several nosocomial infections, illustrating the need for proper infection control practices [51].

## Conclusions

Higher rate of MDR, ESBL and, MBL producing antibiotic resistant *P.aeruginosa* and *A.baumannii* isolates were found in indoor air. Though the current isolates were not identified from patients in this study, the role of contaminated indoor air for the production of ESBL and MBL isolates could play a major role if contact is established. So it is pertinent that their presence should be controlled and antimicrobial stewardship programs should be designed to prevent the further spread of these isolates. ESBL and MBL strains as airborne microbiota are the first finding in Ethiopian that could provide a new insight for antimicrobial stewardship programs and future studies.

### Strength

These bacteria especially *A.baumannii* is the first finding as airborne organism in Ethiopia. Many antibiotics as per the guidelines were tested for these findings and post intervention phase is started after fumigation.

### Weakness

The study didn’t have a plan of post intervention phase by the investigators due to budget limitation even though now the budget was approved for it. The cross sectional nature of this study may increase and decrease the prevalence of the bacteria since patient trafficking, type of patients and other environmental factors like humidity and others may differ in a given day.

## Abbreviations

ATCC: American Type Culture Collection
BAP: Blood agar plates
DR: Delivery room
ESBLs: Extended spectrum beta lactamases
HAI: Health care associated infections
ICU: Intensive care unit
MBLs: Metallo-β-lactamases
MDR: Multidrug-resistant
OR: Operation room
TSA: Tryptic soya agar
WSUTRH: Wolaita Sodo University Teaching and Referral Hospital
